# Annotations

**Published:** 1904-12-31

**Authors:** 


### ANNOTATIONS.

### The New Head of the Army Medical Service.

THE rumour that Sir William Taylor's term of office as Director-General of the Army Medical Service was to be prolonged has been disposed of by the official announcement on Christmas Eve of the appointment to that post of Lieut.-Colonel Alfred Keogh, M.D., C.B. We regard the choice of a comparatively young officer for this high distinction as a welcome recognition of the principle laid down in the report of the War Office Reorganisation Committee that "no consideration except that of special fitness for the duties involved should arise . . . nor need there be any bar to the appointment of an officer of any rank to any post at the War Office, if he possesses the necessary qualifications, experience, and intellectual capacity." How easily such recommendations are accepted in word is as well understood as the outcry which at once arises when an attempt is made to put them in practice, nor is the case unknown in which difficulties have been made by officials who themselves have risen to the posts they occupy by the ignoring of considerations of rank and seniority in their own persons. Born in 1857, the new head of the Army Medical Service passed into the Army in March, 1880, when he was holding a house appointment at the Brompton Hospital for Diseases of the Chest. Upon leaving the Army Medical School he obtained first place from amongst the Indian, British, and naval candidates for commissions, receiving the "Herbert Prize" and the "Martin Memorial Gold Medal" in Tropical Medicine. In March, 1892, he was promoted to the rank of surgeon-major, in March, 1900, to that of lieutenant-colonel, and on July 1st, 1902, he was advanced to the temporary rank of surgeon-general while employed as Deputy Director-General of the Army Medical Service at Headquarters. Surgeon-General Keogh served in South Africa during the war, was in charge of a general hospital, was mentioned in despatches, and awarded the medal with clasp, receiving also the order of C.B. He was complimented by the South African Hospitals Commission for the evidence given before that body, and afterwards served as a member of Mr. Brodrick's Reorganisation Committee for the Army Medical Service and the Army Nursing Service. Personally popular in the department for which he will in future be responsible, Surgeon-General Keogh combines with an intimate knowledge of the needs of the service, the vigour of character which is requisite in order to keep it abreast of the times. Under his direction there is every reason to anticipate that nothing will be left undone to maintain and increase its efficiency.

### Criminal Statistics.

THE criminal statistics (1903) for England and Wales, just issued in the form of a Blue-book, form a volume of very sombre reading. Whilst there is no very impressive change in the figures, it has to be noted that since 1900 there has been a steady increase in the number of persons charged before the Superior Criminal Courts, whereas for the 40 years before that date there had been an almost continuous movement in the opposite direction. The convictions involved no less than 41 death sentences, of which 14 were commuted to penal servitude for life. The reports from the coroners' courts show a decided increase in the number of suicides, an increase which has been steadily advancing for many years. The figures for 1903 were 2,611 males and 869 females, representing a proportion of 10·42 per 100,000 of the population. Forty years ago the proportion of suicides was 6 71. In prosecutions for drunkenness the statistics also show a decided increase over the previous year. A similar statement has to be made in regard to charges for habitual drunkenness, though apparently the report understates the facts on this point, as we are told that "the number of prosecutions has been at times restricted by the limited accommodation in inebriate reformatories." Instances in illustration of this statement must be familiar to all who make a study of police-court records. The total number received into such reformatories was 297, of whom 259 were women, a disparity in the sexes which depends more on the deficient accommodation than on the superior sobriety of the males. According to the police estimate, there were in April 1903 no less than 4,187 habitual criminals at large in England and Wales.

### Cheap Cottages.

THE subject of cheap cottages has been forced on the public attention in a somewhat sensational manner during the last few weeks, and it is certainly one of considerable importance. Possibly it does not appeal to those who have to spend their lives buried in large towns, but even these may remember that the housing of the agricultural classes is a question which closely touches large national interests. It is therefore satisfactory to observe that a scheme is in progress to organise an exhibition of which the principal feature will be model cottages suitable for various localities in England and Scotland. Various prizes will be awarded, including one of £100 which has been offered by the proprietors of *The County Gentleman* for the builder of the best cottage for the sum of £150. The Garden City Company will lend the land for the exhibition, and will promote the sale of those cottages which secure the approval of the adjudicators. In addition to actual buildings, the exhibition will include plans, photographs, designs, appliances, and practical suggestions which may assist in the development of cheap cottage property. It will be interesting to observe to what extent wood will command the confidence of the builders of the model cottages. Though forbidden by many building regulations, and obviously unsuitable for houses in rows or streets in consequence of the danger of fire, wood is claimed by many who speak with experience to be highly suitable for isolated cottages. It is somewhat less expensive than stone or brick, and it makes both a drier and a warmer house. On one large estate on which wooden cottages have been tried they have proved extremely popular with the labourers. In this, and also in many other respects, the proposed exhibition is likely to prove both of interest and value.

